# Episodic and Semantic Autobiographical Memory in Temporal Lobe Epilepsy

**DOI:** 10.1155/2014/157452

**Published:** 2014-12-08

**Authors:** Claudia P. Múnera, Carolina Lomlomdjian, Belen Gori, Verónica Terpiluk, Nancy Medel, Patricia Solís, Silvia Kochen

**Affiliations:** ^1^Epilepsy Center, Neurology Division, Ramos Mejia Hospital, Gral Urquiza 609, C1221ADC Buenos Aires, Argentina; ^2^Center for Clinical and Experimental Neurosciences, Epilepsy , Cognition and Behavior, Institute of Cell Biology and Neurosciences (IBCN), School of Medicine, UBA-CONICET, 2nd Floor, Paraguay 2155, C1121ABG Buenos Aires, Argentina; ^3^National Neuroscience Center, Epilepsy Unit, El Cruce Hospital, Avenue Calchaquí 5401, C1888, Florencio Varela, C1073ABA Buenos Aires, Argentina

## Abstract

Autobiographical memory (AM) is understood as the retrieval of personal experiences that occurred in specific time and space. To date, there is no consensus on the role of medial temporal lobe structures in AM. Therefore, we investigated AM in medial temporal lobe epilepsy (TLE) patients. Twenty TLE patients candidates for surgical treatment, 10 right (RTLE) and 10 left (LTLE), and 20 healthy controls were examined with a version of the Autobiographical Interview adapted to Spanish language. Episodic and semantic AM were analyzed during five life periods through two conditions: recall and specific probe. AM scores were compared with clinical and cognitive data. TLE patients showed lower performance in episodic AM than healthy controls, being significantly worst in RTLE group and after specific probe. In relation to semantic AM, LTLE retrieved higher amount of total semantic details compared to controls during recall, but not after specific probe. No significant differences were found between RTLE and LTLE, but a trend towards poorer performance in RTLE group was found. TLE patients obtained lower scores for adolescence period memories after specific probe. Our findings support the idea that the right hippocampus would play a more important role in episodic retrieval than the left, regardless of a temporal gradient.

## 1. Introduction

Cognitive neuroscience over the years has been trying to elucidate which are the basic mechanisms underlying autobiographical memory (AM). Despite the vast amount of studies performed in this area there is still no consensus on the role of medial temporal lobe (MTL) structures.

Medial temporal lobe epilepsy (TLE) patients provide a unique opportunity to systematically explore different aspects of AM processing considering the involvement of hippocampal structures on seizure onset and the connectivity to local and distal areas of MTL through the neural network related to epileptic spreading [1]. Epilepsy is a “pathologic model” that allows greater opportunities for research in clinical neuroscience than other neurological disorders, like stroke or dementia, in which massive damage of anatomical structures or a degenerative process is observed. An additional advantage is that most of these patients are young adults, whose illness could have begun in childhood, adolescence, or early adult life periods, giving us the chance to compare their performance at different stages. Furthermore, retrieval in this population has not a distinguished base level performance [2] which is central in the assessment of AM.

Two prominent theories argue the role of MTL in the encoding and retrieval of remote AM after consolidation. Briefly, the standard consolidation model (SCM) [3] supports the idea that the hippocampal formation is necessary for encoding episodic and semantic memories, but after consolidation, these memories would become independent of the hippocampus and represented in the neocortex. On the other hand, multiple trace theory (MTT) [4, 5] suggests that MTL structures would be always involved in the retrieval of remote episodic memories and the hippocampus would provide spatial context that could link the details to a “fully elaborated episode memory” [4], and remote semantic memory seems to be independent of the hippocampus, although it initially contributes to its formation and assimilation [5].

Most of the research in AM was conducted in aging, mild cognitive impairment, and degenerative disorders [6–11] while there are fewer investigations in epileptic subjects [2, 12, 13]. The study of AM in TLE would contribute to understanding of the role of MTL and to assessment of daily memory complaints within an ecological approach.

AM is understood as the retrieval of situations lived across lifetime and our own personal experiences that occurred in specific time and space [12, 14, 15] and that are accompanied by the feeling of reliving [16]. The aim of our study was to investigate AM in TLE candidates for surgical treatment analyzing episodic and semantic details throughout different lifetime periods, the association with laterality of epileptic zone (EZ), and other clinical aspects like age of onset of epilepsy, gender, years of formal education, and cognitive status [17].

## 2. Materials and Methods

### 2.1. Participants

Twenty patients with pharmacoresistant TLE candidates for surgery epilepsy were consecutively examined for this study at the Epilepsy Center, Neurology Department, Ramos Mejia Hospital of Buenos Aires, Argentina. Patients aged 18–53 years (*M* = 31.75; SD = 9.63) had an average of 12 years of formal education and were predominantly male (12/8). Only patients with a Full Scale IQ > 70 and without history of psychiatric disorders or other neurological diseases were included. Twenty healthy control subjects were matched to the patients group by age, education, and sex (Table 1).

All subjects gave written informed consent approved by the Institutional Ethics Committee at Ramos Mejia Hospital, which follows the guidelines of the Declaration of Helsinki.

In order to determine lateralization and localization of the epileptogenic zone (EZ), video-EEG monitoring was performed in all patients over 5 days, finding 10 subjects with left EZ (LTLE) and 10 with right EZ (RTLE). An organized seizure activity with a clearly unilateral beginning was found in all patients, with a late propagation to contralateral areas only in 4 LTLE and 3 RTLE. Patients with a bilateral seizure activity from the beginning were not included. A magnetic resonance imaging (MRI) study was conducted for every patient: 19 subjects had hippocampal sclerosis, 10 right and 9 left, and one patient had a left temporal dysembryoplastic neuroepithelial tumor (Table 1).

At the time of the study, all patients were polymedicated with 2-3 AEDs. Only one patient with LTLE had generalized tonic clonic seizures, while five patients (2 left and 3 right) had sporadic secondary generalized seizures.

A neuropsychological assessment was performed according to the CE presurgical protocol [17, 18] and using a *z*-score cutoff of −2. We found a normal memory performance on average of *z*-scores of TLE groups; four of 20 patients (1 RTLE and 3 LTLE) showed deficits in verbal memory measures as observed on the delayed recall of the Rey Auditory Verbal Learning Test. A visual memory deficit was found in 9 of 20 subjects (5 RTLE and 4 LTLE). A deficit in the Boston Naming Test was found in RTLE (6) and LTLE (5) [19] (Table 2).

### 2.2. Autobiographical Interview (AI)

AM was assessed with the Autobiographical Interview [6] translated to Spanish language for our group. We made a pilot study to evaluate comprehension of the questions and to build a list of typical life events adjusted to Argentinean and nearby countries population (e.g., first communion, 15-year birthday (women), wedding day, etc.) keeping the same categories used by Levine et al. [6].

According to the administration instructions [6], subjects were asked to recollect memories from five different time periods (early childhood, teenage years, early adulthood, middle adulthood, and last year), for a total of five memories. Each subject had to choose at least two events for each period and assigned a single “title” per event, so that the examiner randomly chose one title per period. The interview was conducted through three different conditions as follows: (a) “free recall”: subjects described the event chosen without interruption from the interviewer; (b) a “general probe” was used after the free recall when the subject did not understand the task or the event narrated was not clear or did not correspond to the lifetime assessed or to encourage the subject to add more details; (c) after all the events were narrated a “specific probe” was administered for each one of the events and in the same order they were obtained: this probe consists of a semistructured interview to collect additional details. Each one of the lifetime periods was taken into account in the analysis. If subjects were under 30 years of age, they had to recollect two memories instead of one for early adulthood.

Every interview was recorded, transcribed, and segmented in detail or pieces of information. The details were classified as internal-episodic and external-semantic. These were further divided into the following categories: main event, place, time, perceptual details, and thoughts/emotions as internal-episodic information; and repetitions, other details (metacognitive, editorial statements), and factual information as external-semantic information. The information given was segmented and scored to obtain quantitative data following the scoring instructions [6]. One point was given to every detail which was tallied for each category. As was carried out in previous work [6, 12, 20, 21] we add the scores from the general probe condition to those obtained during free recall condition (henceforth found as recall condition).

Additionally, each person assigned a value between 1 and 6 related to how well they visualized the event related, the emotional change produced by the event, the importance given actually and then, and how frequently they talk or think about it. Quantitative ratings were also assigned for episodic information (time, place, perception, and thoughts/emotion) and time integration on a scale of 0 to 3 and episodic richness using a scale extended to 6 points [6]. All memories were transcribed and scored by two independent examiners achieving high interrater reliability.

AI scores were compared between all patients and control group, in RTLE/LTLE versus control, and between LTLE and RTLE. The clinical data and the cognitive status were also analyzed.

### 2.3. Statistical Analysis

Control and TLE group were matched for age, sex, and formal education. For each patient, the raw values of every cognitive test in the neuropsychological battery were normalized to a *z*-score and classified as “deficit” for values less than or equal to −2.

We compared TLE groups versus controls' performance in AI and we analyzed the composite measures considering both the total life span and each period of time. One-way ANOVA, Student's *t*-test, Bonferroni correction post hoc test, Pearson correlation coefficient *r*, Chi-squared test, and logistic regression analysis were used.

All comparisons that were significant at the *P* < 0.05 level were reported. Statistical analysis was carried out using the Statistical Package for the Social Sciences (SPSS version 20).

## 3. Results

### 3.1. Composite Measures of AM

The total number of episodic and semantic details recalled across five life periods was compared for TLE group and control subjects.

**Figure 1:**
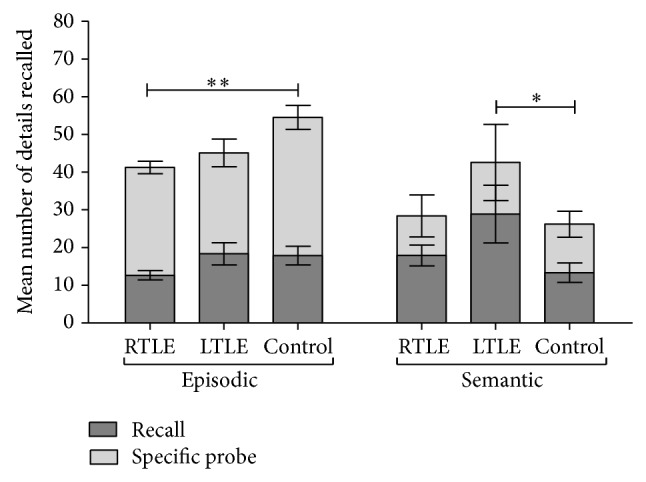
Mean number of episodic and semantic details retrieved during recall (darkest portion of the histogram) and specific probe (lighter portion of the histogram) conditions for each group: RTLE, LTLE, and control. ^*^ *P* < 0.05,  ^**^ *P* < 0.01. Bars indicate the standard error of the mean (SEM).

For episodic details, TLE group scores were lower during recall than control group but we did not find a statistical difference (*P* = 0.286). We found significant differences after specific probe between all patients versus control group (*P* = 0.002) and in RTLE group, retrieving fewer episodic details (*P* = 0.004) compared to controls. When we compared RTLE versus LTLE group, no significant difference was found; however, a tendency to a lower performance for RTLE was sustained (Figure 1).

In relation to semantic details, no differences were found between TLE and controls during either recall (*P* = 0.80) or specific probe (*P* = 0.226). We observed that LTLE group retrieved significantly higher amount of total semantic details (*P* = 0.031) during recall condition but the significant difference disappears after specific probe. No differences were found between RTLE and LTLE (Figure 1).

### 3.2. Specific Autobiographical Retrieval Categories

The number of episodic and semantic details retrieved for individual categories was compared between TLE and control group.

**Figure 2:**
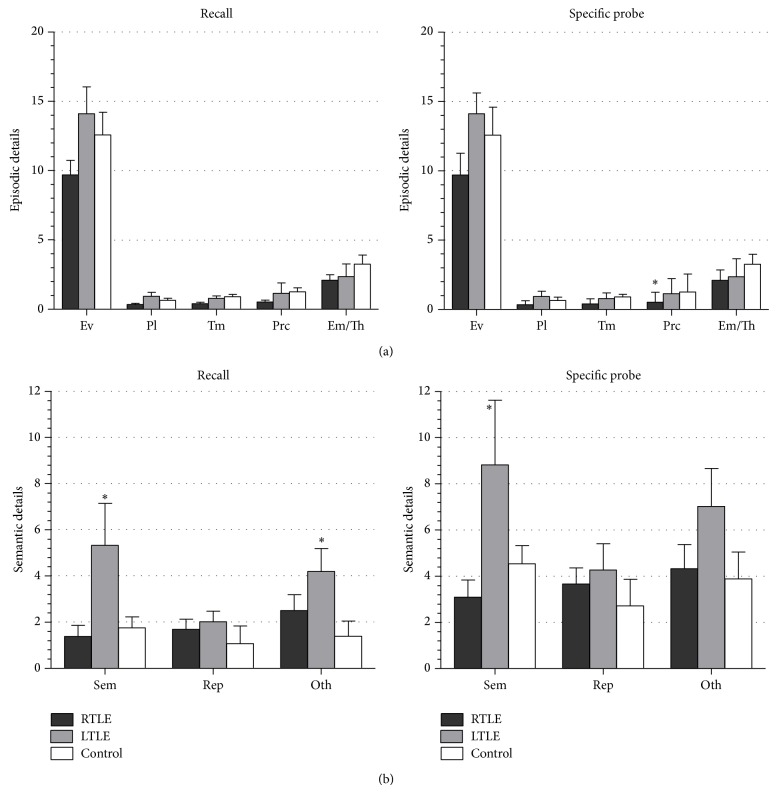
Mean number of (a) episodic and (b) semantic details retrieved for each category during recall and specific probe conditions. All significance levels were at *P* < 0.05. Bars indicate the standard error of the mean (SEM). Ev: event, Pl: place, Tm: time, Prc: perceptual details, Em/Th: emotion/thoughts, Sem: semantic, Rep: repetitions, and Oth: other details.

For episodic details, differences were found between TLE group and control only after specific probe in event details (*P* = 0.020), time details (*P* = 0.022), perceptual details (*P* = 0.006), and emotion/thoughts details (*P* = 0.038). RTLE retrieved lower details for each category compared to controls but significant differences were found only after specific probe for perceptual details (*P* = 0.024). No differences were found between RTLE and LTLE (Figure 2(a)).

For semantic details (Figure 2(b)), TLE scores were statistically different compared to controls only in details classified as “Oth” and during recall condition (*P* = 0.035). LTLE generated significantly higher scores during recall for semantic category factual information (LTLE versus control *P* = 0.027) and the other category metacognitive or editorial statements (LTLE versus control *P* = 0.022), but after specific probe no differences were found. It was observed that LTLE produced more semantic category details than RTLE (recall *P* = 0.041; specific probe *P* = 0.037).

### 3.3. Life Period Analysis of AM

**Figure 3:**
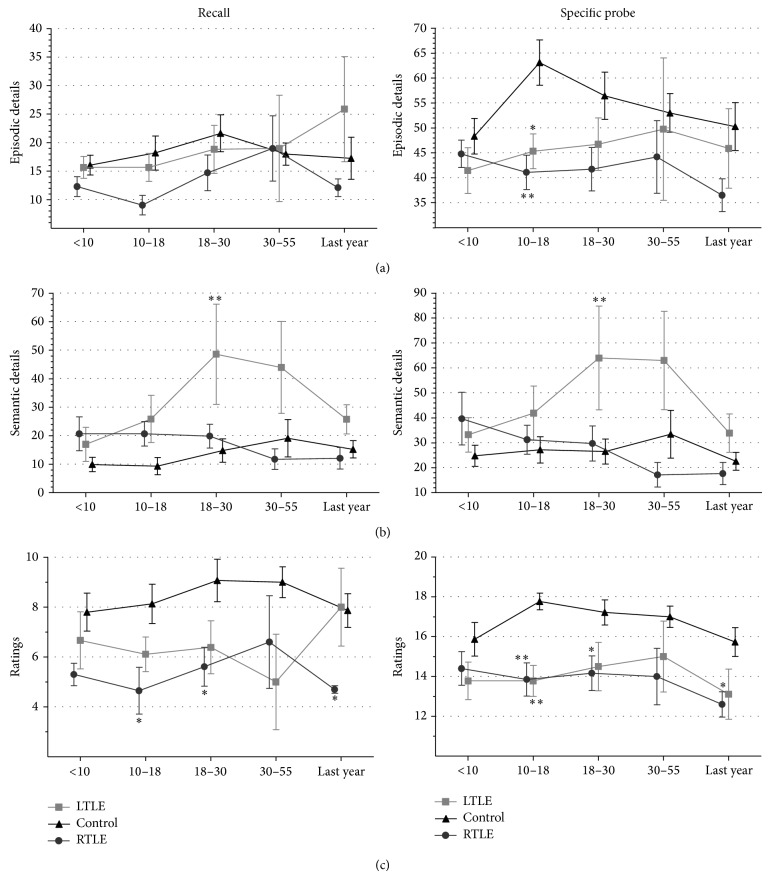
Number of details and ratings retrieved across each one of the life periods, childhood (>10 years), adolescence (10–18 years), early adulthood (18–30 years), and adulthood (30–55 years), and during the last year, for recall (left column) and after specific probe (right column). (a) shows the average number of episodic details, (b) indicates performance for semantic details, and (c) indicates the total score in different rating categories (max = 21). Bars indicate the standard error of the mean (SEM). ^*^ *P* < 0.05,  ^**^ *P* < 0.01.

Figure 3 shows the number of episodic details, semantic details, and rating composite recalled by each group at each of the five life periods. Most remarkable differences were found during the period between 10 and 18 years (adolescence). Both patient groups compared to controls performed poorly for episodic details during each life period but this difference was only statistically significant during the adolescence period and after the specific probe (RTLE *P* = 0.002; LTLE *P* = 0.017) (Figure 3(a)). Semantic details were significantly higher among LTLE compared to controls (recall *P* < 0.001; specific probe *P* < 0.01) and compared to RTLE (recall *P* < 0.05; specific probe *P* < 0.05) (Figure 3(b)) during early adulthood memories.

Ratings composites for recall condition were significantly diminished for RTLE compared to controls not only for adolescence period (*P* < 0.05), but also for early adulthood (*P* < 0.05) and last year (*P* < 0.05) memories. After the specific probe, both RTLE and LTLE obtained lower scores for adolescence period memories (*P* < 0.01) (Figure 3(c)).

### 3.4. Subjective Quality of Autobiographical Memories

**Figure 4:**
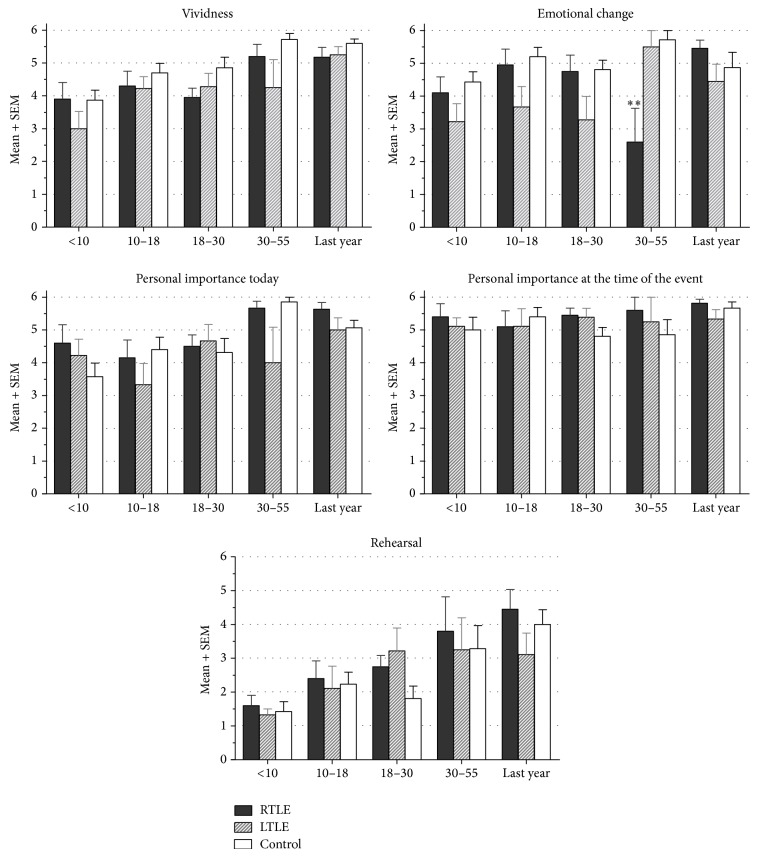
Average of subjective quality ratings for each of the five time periods regarding the vividness of the event, the emotional change experienced, how important the event was at the time of its occurrence and now and how frequently they thought or spoke about it. Bars indicate the standard error of the mean (SEM). ^*^ *P* < 0.05,  ^**^ *P* < 0.01.

Figure 4 indicates ratings assigned by the participants to every memory narrated. An ANOVA showed no effect of group, laterality, or time period (*P* > 0.05) for vividness. When subjects were asked if they experienced an emotional change after the event narrated, it was observed that RTLE reported significantly lower ratings only for adulthood memories compared to controls (*P* < 0.01) and compared to LTLE (*P* < 0.05).

No differences were found when participants were asked how important the event actually is, how relevant it was at the moment of its occurrence, and how frequently they rehearse about it.

### 3.5. AI Scores Correlation with Other Variables

AI scores were compared to neuropsychological test results for each patient. A statistically significant correlation was only found between VIQ and episodic details during recall in RTLE (Pearson correlation coefficient *r* = −0.645). No other significant results were found.

There were no significant correlations between AI scores and age, years of education, age of onset, and disease duration. Our sample does not include cases of recent onset; the duration of the epilepsy was higher than ten years.

## 4. Discussion

Most of previous memory research in epileptic subjects has analyzed different aspects related to type of material, lateralization, and pre- and postsurgical performance [17, 18, 22–25] and a specific group have focused on understanding the quality of epileptic patients AM recollections in comparison to general population [2, 12, 26–28].

Different studies that have used the AI [1, 12] or other tasks to measure AM in TLE [2, 13, 26–30] showed deficits in patients recollections compared to controls. Our findings suggest a significant impaired autobiographical episodic memory only in subjects with right epileptogenic zone. Therefore, the possibility that the right hippocampus would play a more important role in episodic retrieval than the left could be considered. Studies in healthy subjects described a right temporal activation during AM retrieval [31, 32]. In epileptic population it was also found that, after right temporal lobectomy, subjects have a drop in autobiographical episodic memory measures and a poorer performance compared to healthy controls and left temporal lobectomy patients [29, 30]. RTLE episodic deficits in our study become evident only after a specific probing. One possible explanation is that for LTLE and controls the additional questions had triggered a better access to a vast amount of information. For RTLE, the use of “frontotemporal” executive retrieval compensation strategies is not effective which would imply a disruption in that pathway due to the disease. According to Markowitsch [33], the connection between right “anterolateral prefrontal and temporopolar cortices” is critical to recall of past episodic memories.

However, other authors suggest that the left hippocampus is essential to episodic retrieval [28, 34] and proposed the existence of a left-lateralized network which includes not only temporal structures, but also frontal, posterior, subcortical, and cerebellum regions [35]. Within our subjects, LTLE had a lower performance compared to control group, which was not statistically significant.

In relation to episodic detail categories, RTLE presented impaired performance during retrieval after specific probe for perceptual category. No differences were found between RTLE and LTLE. St-Laurent et al. [12] suggest that the impairment in perceptual categories, but not in event categories, might support the idea that hippocampal formation would be necessary “for a rich perceptual re-experiencing” of an episode. AM entails different processes from attention and executive functions to self-reflection, emotion, visual imagery, episodic memory, and semantic processes and according to findings in PET and fMRI studies shows activation in medial and dorsolateral prefrontal, posterior regions and MTL structures, including hippocampus and amygdala [36, 37]. Taking into account previous studies and our findings, we considered that hippocampal structures would be actively involved in episodic remote memories retrieval regardless of a temporal gradient.

Our findings showed no temporal gradient through the five life periods in episodic and semantic AM, as proposed by the consolidation theory [3]. In addition, the reminiscence bump described as a period where “people produce the most memories” [38] usually between 10 and 30 years of age was observed in control group but not in TLE group. Previous studies suggest that the reminiscence bump is related to a specific period of lifetime in which identity and self are being built; therefore autobiographical memories that are highly self-relevant would be preferably encoded [38]. In our study, both right and left TLE patients performed significantly worst for adolescence in episodic AM compared to controls. This finding could be related to important changes that occur during this stage of human development, not only in physical appearance but also in the acquirement of new responsibilities, and has a crucial role in personality characteristics [39]. These changes would be stressful in general, even more for epileptic teenagers that have to deal with discrimination, limited personal choices, and an altered quality of life [40]; thereby learning and retention of new information could be affected [41]. Authors like Berntsen and Rubin [42] suggest that the reminiscence bump would be present only for positive memories but not for negative events.

With regard to semantic AM memory we observed higher performance of LTLE patients on recall condition that disappears after specific probe. In the same way, higher scores in semantic categories, factual information (semantic), and metacognitive statements (the other category) were found which would reflect a compensatory cognitive mechanism [6, 36].

In our work, participants showed no difference with respect to the degree of vividness, personal significance, and rehearsal of their recollections. We could assume that a lesion in MTL structures does not affect these subjects' appreciation. As far as cognitive performance is concerned, we did not observe any relation between verbal or visual memory performance and AI scores that could provide additional data to our results.

Finally, regarding the duration of epilepsy, our interest was to determine its influence in AM recollections but none of the subjects had illness duration fewer than 10 years, so this variable could not be analyzed. The impact of epilepsy across life period recollections was not analyzed either, because the age of onset for the majority of subjects was during childhood.

Our results allow us to provide additional evidence to previous work, of the hippocampal structures involvement in episodic autobiographical memories recollection, particularly the right hippocampus in TLE patients. It is important to consider that one limitation of our study is the relatively small sample size that may contribute to the lack of differences between EZ side groups. For future work, we will compare performance in TLE patients, before and after surgery.

## Figures and Tables

**Table 1:** Demographic and clinical features.

	TLE group		Controls
	Left EZ	Right EZ	
*N*	10	10	20
Age (years)^*^	33,2 (10,97)	30,3 (8,42)	34,07 (11,47)
Education (years)^*^	12,3 (3,09)	12,4 (3,09)	12,93 (2,96)
Sex M/F	7/3	5/5	12/8
Handedness	Right: 10	Left: 2/right: 8	Right: 20
MRI	HS = 9 DNT = 1	HS = 8 HS plus = 2	NA
Age at seizure onset (years)^*^	10,33 (7,69)	8,2 (6,23)	NA
Duration of epilepsy (years)^*^	23,44 (15,42)	22,1 (11,79)	NA
Seizure frequency (per month)	8,57 (10,03)	6,12 (6,93)	NA

^*^Mean (SD). M: male, F: female, HS: hippocampal sclerosis, DNT: dysembryoplastic neuroepithelial tumor, and NA: not applicable.

**Table 2:** Neuropsychological performance of subjects (TLE).

Neuropsychological test	Left EZ			Right EZ		
	Mean (SD)	Range		Mean (SD)	Range	
		Min	Max		Min	Max
WASI: *IQ scores *						
Full Scale IQ (FIQ)	94,66 (12,07)	74	116	80,44 (9,44)	70	96
Verbal IQ (VIQ)	88,37 (14,69)	70	107	78,11 (13,34)	62	95
Performance IQ (PIQ)	100,62 (15,9)	73	126	84,22 (8,46)	71	95
Verbal functioning:*z-scores *						
RAVLT (delayed recall)	−1,36 (1,25)	−2,96	0,29	−0,42 (0,67)	−1,52	0,56
BNT	−3,02 (3,17)	−7,78	1	−2,2 (1,81)	−5,02	0,76
Verbal fluency (phonemic)	−0,90 (0,62)	−2	−0,05	−0,73 (0,9)	−1,68	1,1
Visual functioning:*z-scores *						
RCFT (delayed recall)	−1,41 (1,63)	−3,52	0,56	−1,56 (1,38)	−3,32	0,66

WASI: Wechsler Abbreviated Scale of Intelligence. RAVLT = Rey Auditory Verbal Learning Test. BNT: Boston Naming Test. Phonemic verbal fluency. RCFT: Rey Complex Figure Test. *z*-scores <−2 were considered as deficit.

## Abbreviations

TLE: Temporal lobe epilepsy
AM: Autobiographical memory
AI: Autobiographical Interview.
